# Decompression only versus fusion in octogenarians with spinal epidural abscesses: early complications, clinical and radiological outcome with 2-year follow-up

**DOI:** 10.1007/s10143-022-01805-4

**Published:** 2022-05-10

**Authors:** Pavlina Lenga, Gelo Gülec, Awais Akbar Bajwa, Mohammed Issa, Rod J. Oskouian, Jens R. Chapman, Karl Kiening, Andreas W. Unterberg, Basem Ishak

**Affiliations:** 1 Department of Neurosurgery, Heidelberg University Hospital, Heidelberg, Germany; 2 Department of Neurosurgery, University of Heidelberg, Im Neuenheimer Feld 400, 69120 Heidelberg, Germany; 3 Swedish Neuroscience Institute, Seattle, WA USA

**Keywords:** Surgical decompression, Instrumentation, Octogenarians, Epidural abscess, Osteomyelitis

## Abstract

Despite increased life expectancy due to health care quality improvements globally, pyogenic vertebral osteomyelitis (PVO) treatment with a spinal epidural abscess (SEA) remains challenging in patients older than 80 years. We aimed to assess octogenarians for PVO prevalence with SEA and compare after-surgery clinical outcomes of decompression and decompression and instrumentation. A retrospective review of electronic medical records at a single institution was conducted between September 2005 and December 2020. Patient demographics, surgical characteristics, complications, hospital course, and 90-day mortality were collected. Comorbidities were assessed using the age-adjusted Charlson comorbidity index (CCI). Over 16 years, 35 patients aged ≥80 years with PVO and SEA were identified. Eighteen patients underwent surgical decompression (“decompression group”), and 17 underwent surgical decompression with instrumentation (“instrumentation group”). Both groups had a CCI >6 (mean±SD, 8.9±2.1 vs. 9.6±2.7, respectively; *p*=0.065). Instrumentation group patients had a significantly longer hospital stay but no ICU stay. In-hospital and 90-days mortality rates were similar in both groups. The mean follow-up was 26.6±12.4 months. No further surgeries were performed. Infection levels and neurological status were improved in both groups at discharge. At the second-stage analysis, significant improvements in the blood infection parameters and the neurological status were detected in the decompression group. Octogenarians with PVO and SEA have a high adverse events risk after surgical procedures. Surgical decompression might contribute to earlier clinical recovery in older patients. Thus, the surgical approach should be discussed with patients and their relatives and be carefully weighed.

## Introduction

Despite accelerating improvements in the quality of health care worldwide, pyogenic vertebral osteomyelitis (PVO) with concomitant spinal epidural abscess (SEA) remains a potentially devastating condition. PVO is caused by bacterial infection of the vertebral bodies that can extend into the adjacent intervertebral disc [5], while SEA refers to a collection of purulent material between the dura and the osseous-ligament structures of the spinal cord. In previous decades, the incidence of SEA was very low, ranging from 0.2 to 1.2 cases per 10,000 admissions [3, 8, 14, 25, 26]. However, the numbers are currently rising, with recent estimates ranging from 1.2 to 12.5 per 10,000 admissions [2, 7]. This rapid increase is most likely attributable to the spread of intravenous (IV) drug abuse, an ever-aging population, an increase in spinal surgeries, rising comorbidities such as diabetes mellitus, and the presence of implants providing chronic vascular access [8, 20, 22]. While advanced imaging techniques, efficient IV antibiotics, and advances in surgical techniques have reduced mortality rates, the morbidity remains disturbingly high at 33–47% [8, 20, 22]. Since both symptomatic vertebral osteomyelitis and SEA with neurological deficits are considered surgical emergencies, the main goal of treatment is expedient surgical decompression and antibiotic therapy. Importantly, studies clearly show that the time between the occurrence of neurological symptoms and surgical treatment is pivotal and predictive of neurological recovery [8, 11, 17].

In the case of older and frail patients, the choice of therapy remains controversial, considering perioperative risks associated with patients’ poor general clinical condition. The scarcity of robust evidence concerning the optimal treatment for PVO with SEA in older adults, especially in octogenarians, hampers physicians who must quickly decide whether to perform a surgical procedure.

Owing to the lack of clinical evidence on this topic, we aimed to assess and compare the clinical course and determine morbidity and mortality rates after surgical decompression only versus surgical decompression with instrumentation, exclusively in octogenarians.

## Methods

### Study design and patient characteristics

**Fig. 1:**
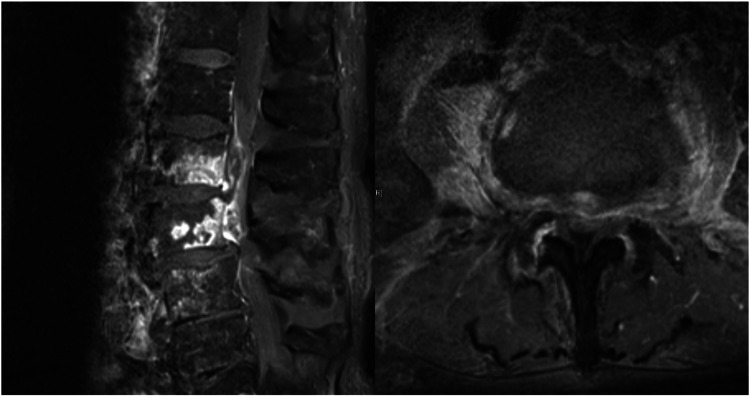
Postcontrast sagittal (**A**) and axial (**B**) magnetic resonance (T1 gadolinium sequence) imaging of dorsal lumbar epidural abscess and early end plate destruction of L2 and L3 of an 85-year-old male patient presenting with lumbar pain and progressive low extremety weakness

We retrospectively evaluated the clinical and imaging data collected from our institution’s database between September 2005 and December 2020. This study was approved by the local ethics committee of our institution (no. 880/2021) and conducted in accordance with the Declaration of Helsinki. The requirement for informed consent was waived because of the retrospective nature of the study. Patients aged ≥80 years with PVO and SEA across the thoracic and lumbar spine were consecutively enrolled. The diagnosis was based on magnetic resonance imaging (MRI) (Fig. 1). Spine stability was examined by findings of computed tomography (CT). Exclusion criteria were as follows: <80 years old, had a concurrent intracranial or cervical pathology, the requisite data were unavailable. Exclusion criteria because of spinal instability based on CT findings were as follows: bony deconstruction resulting in kyphosis or subluxation of the vertebral column, vertebral collapse of more than 50% or bone necrosis, complete loss of disc height. The electronic medical records were assessed to obtain patient demographics, comorbidities, American Society of Anesthesiologists (ASA) scores, duration of surgery, number of treated spinal levels, perioperative and postoperative complications, hospital length of stay (LOS), intensive care unit (ICU) stay, readmissions, reoperations, and mortality. Comorbidities present before surgery were assessed using the age-adjusted Charlson comorbidity index (CCI) [10, 13]. The CCI was calculated for each patient and classified as follows: no comorbidity (CCI = 0), minimal comorbidity (CCI = 1 or 2), moderate comorbidity (CCI = 3–5), or severe comorbidity (CCI > 5). The pretreatment neurological condition was assessed using the motor score (MS) of the American Spinal Injury Association (ASIA) impairment grading system (MS = 0, no muscle strength; MS = 100, healthy). The posttreatment MS was obtained from the last clinical encounter documented.

Routine clinical and radiological follow-up examinations were performed before discharge and at 3 months after surgery. The final follow-up varied between 3 and 72 months postoperatively. Standard radiographs in the anteroposterior and lateral views were obtained to evaluate screw position and fusion rate.

### Surgical procedures

Patients were allocated into two groups: (1) surgical decompression only (decompression group) and (2) surgical decompression with spinal instrumentation (instrumentation group). Furthermore, as an additional tool to classify our cases, we applied the classification system of Pola et al. [23]. According to the system, our cases belonged to the type 3.C., meaning epidural abscess without instability signs and suggesting the performance of surgical decompression. Since the interobserver variability of this score showed moderate to substantial agreement (Fleiss *κ* value 0.6–0.63) [6] and many confounders need to considered in case of octogenarians, the decision-making was guided by patients’ individual characteristics. When deciding for decompression alone or decompression with instrumentation, we evaluated the location of the abscess. In dorsally accessible abscess, the minimal invasive drainage via laminotomy was utilized, while ventrally located granulated tissue an instrumentation approach was considered, as previously described (Camino Willhuber et al., 2021). The decision-making was mainly guided by presenting neurological status (MS), the concomitant underlying pathologies, the extent of the pathology, and the discretion of an experienced treatment team consisting of neurosurgeons, neuroradiologists, and anesthesiologists. An important deciding role also played the risk of secondary instability after a simple decompression since previous studies have reported significant higher reoperation rates in patients initially opted for a decompression surgery [16, 18].

Considering all that points, the attending surgeon decided for decompression only or decompression with instrumentation. A CT-based point-to-point navigation system was used to perform spinal instrumentation, as previously described by our study group [15].

In line with our institutional treatment protocols, blood samples or intraoperative cultures were collected prior to administration of IV antibiotics. Thereafter, IV antibiotics were initiated immediately. After identifying the bacterial specimens, the choice of IV antibiotics was adapted to reflect the antibiogram results.

### Statistical analysis

Categorical variables are presented as numbers and percentages. Continuous variables are presented as means ± standard deviations and were verified as normally distributed using the Shapiro–Wilk test. Baseline characteristics, duration of surgery, number of treated spinal levels, perioperative and postoperative complications, LOS, ICU stay, readmissions, reoperations, and mortality were compared groupwise using independent *t*-tests for continuous variables and chi-squared tests for categorical variables. The Wilcoxon rank test was applied to evaluate changes in C-reactive protein (CRP), leukocytes, and neurological status (as MS) of each group at discharge. Because these procedures are uncommonly applied in patients aged >80 years, and because our sample was relatively small, we could not perform a multivariate analysis to adjust for potential confounders. A *p*-value ≤ 0.05 was set as statistically significant.

## Results

### Patient demographics and baseline characteristics

Over a period of 15 years, 35 patients ≥ 80 years of age diagnosed with PVO and SEA were enrolled in the study. Our sample had an overall mean age of 82.2 ± 1.3 years, with a predominance of men (*n* = 24, 68.6%). Eighteen patients were allocated to the decompression group and 17 to the instrumentation group, respectively. No significant intergroup differences in comorbidities were observed. Both groups had a CCI > 6, indicating a poor prior baseline reserve (8.9 ± 2.1 vs. 9.6 ± 2.7, respectively; *p* = 0.065). No significant differences were observed between the groups regarding the extent or location of the SEA. The lumbar spine was the most amenable region in both groups, followed by the thoracic region. Patients in both groups presented with a lumbar abscess with an extension to at least two levels. Blood infection parameters were relatively high, with CRP levels 173 ± 15.8 mg/L in the surgical decompression group and 150.3 ± 14.6 mg/L in the instrumentation group (*p* < 0.005). No differences were observed in motor impairment as measured by ASIA scoring (Table 1).

**Table 1:** Baseline patient characteristics

	Decompression *N*^a^ = 18	Instrumentation *N* = 17	*p*-value
Age, y (mean, SD^b^)	82.8 (1.2)	81.6 (1.6)	0.195
Sex (*n*^c^, %)			0.328
Male	11 (61.1)	13 (76.5)	
Female	7 (38.9)	4 (23.5)	
BMI^d^, kg/m^2^ (mean, SD)	27.4 (5.0)	26.8 (3.7)	0.610
Comorbidities			
Age-adjusted CCI^e^ score (mean, SD)	8.9 (2.1)	9.6 (2.7)	0.065
Arterial hypertension (*n*, %)	14 (77.8)	13 (76.5)	0.927
Myocardial infarction (*n*, %)	1 (5.6)	5 (29.4)	0.061
Coronary heart disease (*n*, %)	12 (66.7)	9 (52.9)	0.407
Atrial fibrillation (*n*, %)	4 (22.2)	7 (41.2)	0.227
Heart failure (*n*, %)	6 (33.3)	5 (29.4)	0.803
COPD^f^ (*n*, %)	3 (16.7)	7 (41.2)	0.109
Diabetes mellitus type II (*n*, %)	5 (29.4)	8 (47.1)	0.290
Renal failure (*n*, %)	9 (50.0)	9 (52.9)	0.862
Liver disease (*n*, %)	2 (11.1)	3 (17.6)	0.339
Gastrointestinal ulcer (*n*, %)	3 (16.7)	4 (23.5)	0.618
TIA^g^/stroke (*n*, %)	0 (0.0)	2 (11.8)	0.122
Malignancy (*n*, %)	2 (11.1)	3 (17.6)	0.530
Dementia (*n*, %)	1 (5.6)	1 (5.9)	0.932
Previous spinal surgery (*n*, %)	4 (22.2)	3 (17.6)	0.671
ASA^h^ class (*n*, %)			0.737
II	3 (16.7)	2 (11.8)	
III	12 (66.7)	10 (58.8)	
IV	3 (16.7)	3 (17.6)	
V	0 (0.0)	1 (5.9)	
Localization of epidural abscess (*n*, %)			0.870
Thoracic	8 (44.4)	8 (47.1)	
Thoracolumbar	2 (11.1)	1 (5.9)	
Lumbar	6 (33.3)	7 (41.2)	
Lumbosacral	2 (11.1)	1 (5.9)	
CRP^i^ level, mg/L (mean, SD)	173.4 (15.8)	150.3 (14.6)	0.683
Leukocytes, count/L (mean, SD)	11.9 (5.6)	12.2 (4.8)	0.946
Preoperative neurological deficit (*n*, %)	16 (88.9)	15 (88.2)	0.330
Preoperative MS^j^ score (mean, SD)	79.4 (17.1)	77.9 (25.9)	0.386

^a^Group size; ^b^standard deviation; ^c^number of patients; ^d^body mass index; ^e^Charlson comorbidity index; ^f^chronic obstructive pulmonary disease; ^g^transient ischemic attack; ^h^American Society of Anesthesiologists; ^i^C-reactive protein; ^j^motor score of the American Spinal Injury Association grading system

### Surgical characteristics and clinical scores

As shown in Table 2, the surgical duration of patients in the instrumentation group was significantly longer than that of those in the decompression-only group (203.5 ± 104.1 min vs. 132.8 ± 89.7 min; *p* = 0.007). In addition, the number of operated levels was significantly greater in patients with instrumentation than in patients with mere surgical decompression (3.7 ± 1.6 levels vs. 1.9 ± 1.2 levels; *p* < 0.001). Notably, while patients of the instrumentation group had significantly longer hospital LOS, the ICU stay was similar between both groups. Concerning the in-hospital mortality, no significant differences were obtained. The 90-day mortality rate was 23.5% in patients undergoing instrumentation versus 5.6% in patients with surgical decompression only; however, this difference did not reach statistical significance (*p* > 0.05). Moreover, in both groups, infection levels and neurological status showed significant improvement at discharge. Overall, the mean follow-up period was 26.6 ± 12.4 months, and no additional surgery was necessary due to secondary instability. Furthermore, no screw loosening or displacement was seen on radiographs.

**Table 2:** Comparison of surgical characteristics and clinical course between groups

	Decompression *N*^a^ = 18	Instrumentation *N* = 17	*p*-value
Surgical duration, min	132.8 (89.7)	203.5 (104.1)	0.007*
No. of levels decompressed/fused	1.9 (1.2)	3.7 (1.6)	<0.001*
Hospital stay, days	8.9 (6.2)	14.8 (10.4)	0.020*
ICU^b^ stay, days	3.1 (2.3)	4.2 (3.1)	0.405
Mortality			
In-hospital (*n*^c^, %)	1 (5.6)	2 (11.8)	0.512
90-day (*n*, %)	1 (5.6)	4 (23.5)	0.129
Post CRP^d^	108.7 (93.6)	116.6 (78.9)	0.357
Delta CRP	−64.7 (68.2)	−33.7 (59.7)	0.193
Post leukocytes	10.0 (5.1)	10.6 (3.5)	0.760
Delta leukocytes	−1.9 (2.5)	−1.6 (5.4)	0.858
Post MS^e^	83.6 (15.5)	80.0 (24.2)	0.935
Delta MS	4.2 (8.4)	2.1 (1.5)	0.067

Except where otherwise indicated, quantities are mean (SD)

*Significant difference; *Post*, after surgery; *Delta*, difference between presurgical and postsurgical values

^a^Group size; ^b^intensive care unit; ^c^number of patients; ^d^C-reactive protein; ^e^motor score of the American Spinal Injury Association grading system

### Complications

We observed a significant intergroup difference only in the occurrence of pleural effusion, with higher rates in the instrumentation group (23.5% vs. 0.0%; *p* = 0.029). A detailed breakdown of all recorded complications is provided in Table 3. *Staphylococcus aureus* was detected in about half of the patients in the instrumentation (70.6%) and decompression (44.4%) groups, either in blood or intraoperative samples. The next most frequent pathogen in both groups was *Escherichia coli* (instrumentation: 16.7% vs. decompression groups: 11.8%), followed by *Enterococcus* sp. (instrumentation: 11.1% vs. decompression groups: 5.9%), *Pseudomonas aeruginosa* (instrumentation: 5.6% vs. decompression groups: 5.6%), and multiple pathogens (instrumentation: 5.6% vs. decompression groups: 5.6%) respectively. No pathogens were identified in 16.7% of the decompression group and 5.9% of the instrumentation group.

**Table 3:** Occurrence of adverse events

	Decompression *N*^a^ = 18	Instrumentation *N* = 17	*p*-value
Deep wound infection	1 (5.6)	2 (11.8)	0.512
Acute respiratory failure	1 (5.6)	1 (5.9)	0.219
Acute heart failure	1 (5.6)	2 (11.8)	0.296
Acute renal failure	1 (5.6)	0 (0.0)	0.324
Septic shock	0 (0.0)	2 (11.8)	0.967
Pneumonia	2 (11.1)	5 (29.4)	0.228
Pleural effusion	0 (0.0)	4 (23.5)	0.029*
Ileus	0 (0.0)	1 (5.9)	0.296
Urinary tract infection	0 (0.0)	1 (5.9)	0.296

All data are number of patients (%)

*Significant difference

^a^Group size

### Comparison of outcomes

In a second-stage analysis, we examined the clinical outcomes in relation to the treatment strategies. After both types of surgery, a significant improvement was observed in blood infection parameters and neurological status at discharge compared to baseline measurements solely in the decompression group. In contrast, in the instrumentation group, statistical significance was reached only concerning the CPR levels. The clinical outcomes of both groups are provided in Table 4.

**Table 4:** Comparison between baseline (before surgery) and discharge

	Decompression Baseline *N*^a^ = 18	Decompression Discharge *N* = 18	*p*-value	Instrumentation Baseline *N* = 17	Instrumentation Discharge *N* = 17	*p*-value
CRP^b^	173.4 (15.8)	83.6 (15.5)	0.003*	150.3 (14.6)	116.6 (78.9)	0.031
Leukocytes	11.9 (5.6)	10.0 (5.1)	0.007*	12.2 (4.8)	10.6 (3.5)	0.113
MS^c^	79.4 (17.1)	83.6 (15.5)	0.004*	77.9 (25.9)	80.0 (24.2)	0.170

All data are presented with mean (SD)

*Significant difference

^a^Group size; ^b^C-reactive protein; ^c^motor score of the American Spinal Injury Association grading system

## Discussion

Spinal epidural abscess is well known to be a devastating condition and is associated with high rates of neurological disability, ranging from 15 to 27%, and mortality, ranging from 5 to 16% [8, 9, 25]. Surgical procedures such as decompression are of paramount importance for obtaining a good clinical outcome and a satisfactory recovery. However, robust evidence comparing surgical strategies such as decompression only versus decompression with instrumentation in older patients remains scarce, especially in those aged ≥ 80 years, who are exceptional because of their poor baseline reserve.

### Summary of findings

To our knowledge, this is the first systematic analysis describing surgical strategies in octogenarians with PVO and concomitant SEA. The current study examined the clinical vignette, the neurological condition, the surgical characteristics, and the clinical course of this scarce disease with the aim of assessing morbidity and mortality rates. Interestingly, we found no significant differences between patients undergoing decompression only and those undergoing decompression with instrumentation regarding the following parameters: comorbidities, infection levels as determined by laboratory parameters (CRP and leukocytes), and grade of disability as defined by the MS score. Notably, patients undergoing pedicle screw fixation and surgical decompression had substantially longer surgical time and duration of hospitalization but no longer ICU stays. Also of note, among patients who opted for a surgical decompression procedure, we observed a significant improvement in the infection parameters and motor function at discharge, while in the instrumentation group, only the reduction in CRP levels reached statistical significance.

### Literature review

Kim et al. retrospectively studied 16 patients aged > 65 years with PVO and SEA. Almost all patients (87.5%) underwent surgical decompression only [17]. Three patients were older than 80 years. Conversely, in the present study, we examined 35 patients older than 80 years, of whom 18 underwent surgical decompression only, and 17 underwent surgical decompression with instrumentation. Compared to Kim et al., who found a relatively low morbidity rate and a CCI as high as 4 in only 3 patients, our study cohort in both groups presented severe comorbidities with a mean CCI of 9.0 or greater [17]. Kim and colleagues did not report on surgical duration, ICU stay, or hospital stay and observed a higher mortality rate after surgery (31.3%) [17]. In contrast, the in-hospital mortality in our group undergoing surgical decompression only was fundamentally lower (5.6%). The substantial discrepancy between the mortality rates might be attributable to the presence of delayed surgeries in the cited study, which were performed 40 days after the diagnosis. After completing the diagnostic work-up, our patients were operated on in less than 24 h with concurrent administration of IV antibiotics which might explain our low mortality rates even in older patients with multiple comorbidities.

It is well known that the C-reactive protein (CRP) is elevated in more than 90% of cases with epidural abscess and PVO and is considered as the most specific marker for treatment response [19]. Alton et al in their retrospective analysis of 62 patients with cervical epidural abscess found comparable rates of infection levels (CRP, count of leukocytes) between medically and surgically treated patients. The failure of medical therapy was based mainly on the progression of neurological deficits rather than laboratory parameters like CRP [1]. It is worth noting that in case of octogenarians, the CRP levels can be also elevated due to the poor prior baseline history [32]; hence, its specifity and sensitivity might be decreased. In keeping with these data, our findings also suggest comparable infection levels between both groups. The decision-making was guided primarily not by laboratory parameters but by clinical judgment, imaging findings, and surgeons’ experience. Notwithstanding, laboratory parameters should be closed monitored since a steep increase might be a herald of a progressive disease.

Noteworthily, patients undergoing surgery showed a trend towards improvement of the motor function, while the CRP levels decreased significantly, thus indicating the start of recovery after spinal infection. No significant differences were shown between both groups in terms of the laboratory infection parameters at discharge or the motor function, hence supporting that both techniques should be considered when deciding for surgery. However, the advantages and shortcoming of each method should be thoroughly discussed and considered. Lee et al. in their retrospective analysis of 47 patients with a mean age of 56 years did not find any differences concerning the neurologic function as well [18]. Here, it is important to accentuate that in the aforementioned study, laboratory parameters were not analyzed. We strongly believe that the significant motor improvements of the decompression group at discharge are attributable to shorter operation times and better neurological status after surgery. A thorough neurologic examination in long-term follow-up should be conducted to quantify the recovery of such cases.

Another retrospective analysis of 135 patients with PVO and SEA aged 18–88 years found that surgery led to significant improvements in neurological condition compared to medication alone [27]. About half of the cases were converted to surgical decompression and the other half to surgical decompression with instrumentation. However, the authors did not distinguish the outcomes of the two surgical approaches; thus, they broadly claimed that surgical treatment might be a critical pillar when treating patients with infections [27]. In another analysis of 60 patients with cervical SEAs and a mean age of 53 years, Alton et al. concluded that early surgery might be the key to the concurrent improvement of the infection and neurological statuses. Surprisingly, comorbidities did not predict treatment failure [1]. In agreement with these findings, Patel et al. compared medication versus surgical therapy in patients with PVO and SEA and advocated for early surgery based on an improvement of the MS by at least 3.4 points, also finding that diabetes mellitus, elevated CRP, and leukocytes were significant predictors of medical failure [21]. Based on the previous studies, the first-line therapy for such a devastating illness at our institution was surgery and not conservative management.

In a retrospective analysis of 40 patients with spinal abscess, Du et al. found that greater age (> 60 years) and the presence of comorbidities such as diabetes mellitus, respiratory, renal, or tumor diseases, and thrombocytopenia might significantly contribute to higher mortality rates evaluated 30 days postsurgery. They did not determine the surgical procedure [12]. Their overall 30-day mortality was 3.7%, comparable to those found by Darouiche et al. and Vakili et al. [8, 31]. Interestingly, the most frequently reported complications were septic shock, cardiac arrest, and pneumonia which were associated with mortality. In contrast, our mortality rates were substantially higher at discharge (8.7%) and at 90 days postsurgery (14.3%). This phenomenon might be attributable to our patient subset consisting only of patients older than 80 years and having many compounding factors (CCI > 6). Although each patient was postoperatively admitted to the ICU to prevent or treat postoperative complications, death was inevitable in some cases. Surprisingly, we found that older patients undergoing surgical decompression with instrumentation do not have a significantly greater mortality risk than surgical decompression alone. One explanation might be that the consistent use of spinal navigation in our surgical routine might be key for decreasing or even avoiding intraoperative complications and prolonged surgeries, thereby diminishing the rate of unanticipated postoperative events.

Hospital-acquired infection (HAI) is a serious complication of the modern health care system. As the elderly population increases globally, the number of geriatric patients admitted to the ICU has also increased strikingly. For instance, patients over 65 years account for 42–52% of those in the ICU [28]. Risk factors predisposing HAI contain both extrinsic factors such as surgery, medication, and hospital stay and intrinsic factors such as age, sex, comorbidities, and immunosuppression; thus, octogenarians are more prone to infections due to their reduced immunological competence and complications of their chronic illness [28, 33]. Previous evidence suggest that elderly patients are at a significant increased risk (risk ratio of at least 2.5) for the occurrence of urinary tract infection, respiratory infections, and septicemias [28]. Notably, elderly patients experience substantially more infection HAI for each hospitalization day especially after day 7 [28]. In line with the aforementioned studies, we did show that patients undergoing instrumentation reveal higher rates of medical complications when compared to the ones of the decompression group only. For instance, septic shock and respiratory complications were present in 11.8% and 58.8% of patients in the instrumentation groups, respectively, while none of the patients in the decompression group experienced a septic shock and only 16.7% suffered from respiratory complications. Therefore, when deciding a surgical procedure in the elderly, all these factors should be considered to alleviate such risks.

The safety of spinal instrumentation in the setting of infection might have been viewed with some controversy in the past few decades. In case of unresponsiveness to conservative treatment, a surgery aims to eradicate the infection focus, restore the neurological function, relieve the pain, and protect or restore spinal alignment. Rayes et al. in their retrospective study on 37 patients with spinal infection performed solely instrumentation surgery. In almost 80% of the cases, a successful arthrodesis was observed, while failure of implants did not occur in any case [24]. In another retrospective study, Schomacher et al. stated that graft-assisted instrumentation such as polyethereketone cages or titan cages can be utilized without the risk of reinfection with comparable fusion rates [29]. Furthermore, a recent study showed 0% rate of recurrence in patients with spinal infection treated with instrumentation or fusion [30]. Therefore, as evidence supporting the safety of instrumentation in face of spinal infection increases, after meticulous interdisciplinary discussion of every case, 17 patients of study cohort opted for instrumentation and no implant failure was observed over a 2-year follow-up. Consequently, we strongly believe that the instrumentation might be a suitable surgical technique even in octogenarians.

In the current context, whether to perform surgical decompression alone or to supplement decompression with instrumentation in cases with active spinal infection is still a subject of debate. Albeit decompression is the primary goal for the treatment of SEA, the concern of surgeons for the occurrence of secondary spinal instability led them to combine decompression with spinal stabilization (Saviteer et al., 1988). Dietz et al. described in their retrospective analysis based on claims data from 2662 patients with spinal infections including SEA and PVO that decompression with instrumentation resulted in better outcomes in terms of re-admission and re-operation. Herein, it is important to highlight that their cohort consisted mainly of younger individuals with an overall mean age of 56.6 years with a low rate of comorbidities, which might have influenced both decision-making and the natural course of the disease. Nevertheless, the authors advocated that the decision-making was mainly guided by the surgeon’s experience. Interestingly, another study comparing patients undergoing decompression alone vs. decompression and instrumentation for spinal infections stated that the choice of the surgical procedure was determined by the attending neurosurgeon treating the patient at each time due to the lack of clear consensus [18]. In conjunction to our findings, Lee et al. found no significant differences concerning the neurological outcomes or the complications rates between both groups, while a re-operation because of secondary instability or progressive axial pain was performed in approximately 50% of patients in the decompression group. In line with these findings, Karadimas et al. also observed significantly higher reoperation rates in the decompression group when compared to decompression and concomitant instrumentation [16]. Furthermore, the merit of previous classification scores is limited due to the moderate interobserver agreement and the questionable usefulness in clinical practice (Camino Willhuber et al., 2021). It seems somehow surprising that in our study cohort, no re-operation was needed. Potential explanations might be the meticulous interdisciplinary study of both clinical and imaging data in terms of instability which was discussed by surgeons, neuroradiologists, and anesthesiologists aiming to determine the most beneficial concept for each individual. Finally, a quite frequent phenomenon observed in older adults is the progressive degeneration of the spine as well as the spontaneous fusion, which might have contributed to higher rates of stability [4].

### Strengths and limitations

The main strength of the current study is that we are the first to examine the outcomes of octogenarians undergoing surgery for SEA. However, this study has some limitations. First, we examined a relatively small cohort of patients. Nevertheless, since there is a lack of robust evidence of the clinical course of such a devastating disease in older individuals, we believe that our findings greatly clarify the clinical picture. Second, the minimum follow-up period of 12 months was relatively short; by gathering long-term data, other relevant findings not captured in the current study might have been revealed. Third, as this is a retrospective study, selection bias may have been present. Larger studies might be needed to elucidate potential candidates for non-operative management with antibiotic therapy only.

### Implications and future directions

Based on our observation of a fast recovery in patients undergoing surgical decompression only, one might argue that surgical decompression might be the key treatment strategy for the patients studied here. However, we believe a multidisciplinary approach primarily involving experienced spine surgeons is needed to determine an adequately individualized therapy for frail older patients with severe baseline histories. Larger studies are needed to investigate the trends observed in our patient population further.

## Conclusions

Due to a steadily increasing average life expectancy, spine surgeons frequently encounter older patients requiring surgical therapy. Our results show that surgical decompression only and decompression with instrumentation can be considered safe treatments strategies for patients with SEA older than 80 years. It seems that older patients undergoing only surgical decompression might recover neurologically and clinically more quickly than patients who had additional instrumentation. However, the surgical treatment should be clearly discussed with the patient and their relatives. We hope this study will serve as a basis for developing a consensus on the best treatment strategy for frail patients confronting such a devastating illness.

## Data Availability

The datasets generated during and/or analyzed during the current study are available from the corresponding author on reasonable request.
